# Whole Transcriptome Analysis of Male and Female Northern Pike (*Esox lucius*)

**DOI:** 10.3390/biology15120898

**Published:** 2026-06-08

**Authors:** Junjie Zhang, Zhelan Wang, Qian Xiao, Xinan Fu, Sitong Li, Shuhan Chen, Yang Cao, Xuefei Zhao, Yu Zhang

**Affiliations:** 1 College of Life Sciences, Xinjiang Agricultural University, Urumqi 830052, China; m15286133601@163.com (Z.W.);; 2 Xinjiang Key Laboratory for Ecological Adaptation and Evolution of Extreme Environment Organisms, College of Life Sciences, Xinjiang Agricultural University, Urumqi 830052, China; 3 Xinjiang Characteristic Aquaculture Research Center, Xinjiang Agricultural University, Urumqi 830052, China; 4 Modern Fisheries Industry College, Xinjiang Agricultural University, Urumqi 830052, China

**Keywords:** RNA, sex differentiation, sex-biased genes, regulatory pathways

## Abstract

The northern pike (*Esox lucius*) is an economically important cold-water fish species in northern China. It exhibits pronounced sexual dimorphism, yet the molecular mechanism underlying its sex differentiation remains unclear, which hinders the development of aquaculture. Whole-transcriptome sequencing is a powerful approach for screening sex-related genes; however, no such study has been reported for this species to date. In this study, gonadal tissues from three female and three male *E. lucius* were collected for whole-transcriptome sequencing. A total of 14,941 differentially expressed messengerRNAs, 119 differentially expressed microRNAs, 229 differentially expressed circularRNAs, and 2055 differentially expressed long non-codingRNAs were identified. Functional enrichment analysis revealed that the differentially expressed genes were significantly enriched in pathways closely associated with sex differentiation, such as steroid hormone biosynthesis and oocyte meiosis. Several key sex-biased genes were identified, including female-biased genes (*FANCL*, *DDX5*, *SRSF5B*) and male-biased genes (*STAR*, *FDX1B*, *ITGA2B*). Furthermore, a competing endogenous RNA (ceRNA) regulatory network involving dre-miR-107b was constructed, which may represent a candidate for further investigation into sex differentiation in *E. lucius*. This study provides the first comprehensive whole-transcriptome dataset of female and male gonads in *E. lucius*, identifies key sex-biased genes and core pathways involved in its sex differentiation, and thereby identifies the dre-miR-107b-centered ceRNA network and key sex-biased genes (*FANCL*, *DDX5*, *SRSF5B*, *STAR*, *FDX1B*, *ITGA2B*) as core molecular players in sex differentiation of this species.

## Simple Summary

The northern pike (*Esox lucius*) is an economically important cold-water fish in northern China. It exhibits significant growth differences between males and females, but its sex differentiation mechanism remains unclear, which hinders the development of aquaculture. In this study, we performed whole-transcriptome sequencing on the gonads of three females and three males. A large number of differentially expressed messenger RNAs, microRNAs, circular RNAs, and long non-coding RNAs (lncRNAs) were identified. Functional analysis showed that these differentially expressed genes are significantly enriched in pathways closely related to sex differentiation, such as steroid hormone biosynthesis and oocyte meiosis. Several female-biased and male-biased key genes were identified. Furthermore, a competing endogenous RNA (ceRNA) regulatory network centered on dre-miR-107b was constructed, which may serve as a candidate for further study on sex differentiation in the northern pike. This study provides the first complete whole-transcriptome dataset of female and male gonads in this species, laying a theoretical foundation for understanding its sex differentiation mechanism.

## 1. Introduction

Sex differentiation is a core biological event during fish development, with complex regulatory mechanisms involving the synergistic action of genes, messenger RNAs (mRNAs), long non-coding RNAs (lncRNAs), circular RNAs (circRNAs), microRNAs (miRNAs), and signaling pathways. As a direct manifestation of sex differentiation, the molecular basis of sexual dimorphism is precisely the differential expression of genes and non-coding RNAs between male and female individuals [1,2,3]. With the rapid development of high-throughput sequencing technologies, whole-transcriptome analysis has become an important tool for investigating the molecular mechanisms of sexual differentiation in fish. This approach has been widely applied in several fish species, such as half-smooth tongue sole (*Cynoglossus semilaevis*), large yellow croaker (*Larimichthys crocea*) and Yellow River carp (*Cyprinus carpio haematopterus*), and has successfully screened a number of key genes and regulatory pathways involved in sex differentiation [4,5,6].

Among the main mechanisms involved, the steroid hormone biosynthesis pathway, the mitogen-activated protein kinase (MAPK) signaling pathway, and the Wnt signaling pathway stand out as playing central roles in the sexual differentiation of teleost fish [7,8,9]. In addition, genes such as *dmrt1*, *sox9*, and *cyp19a1* are central genes for sex determination [10,11,12]. For example, whole-transcriptome analysis in zebrafish (*Danio rerio*) identified 3604 differentially expressed genes between male and female gonads, including *foxl2* and *cyp19a1a* in females and dmrt1 and amh in males, which were significantly enriched in the steroid hormone biosynthesis pathway [13]. This pattern is directly related to gonadal differentiation, as also described by Li et al. [14].

In addition to coding genes, the competitive endogenous RNA (ceRNA) network acts as an important epigenetic mechanism in the regulation of gene expression. In this network, lncRNAs, circRNAs, and mRNAs compete for miRNA binding sites, thereby indirectly modulating target genes. Evidence indicates that this network actively participates in sexual differentiation and dimorphism in fish [1,15]. In *C. semilaevis*, the circRNA circdmrt1 can adsorb miRNAs and consequently regulate gsdf expression, participating in signaling pathways associated with the regulation of male development [1]. These findings provide new perspectives on the epigenetic mechanisms underlying sexual differentiation and dimorphism in fish.

Despite these advances in different species, the transcriptional mechanisms involved in the sexual differentiation of the northern pike (*Esox lucius*) remain poorly understood. The species is widely distributed in freshwater environments of North America and northern Eurasia, occurring mainly in the Irtysh River basin in Xinjiang, China. In addition to representing an important regional economic resource, *E. lucius* is one of the main species in local aquaculture [16,17]. *E. lucius* exhibits marked sexual dimorphism in growth, with females having larger body size, later sexual maturity, and longer lifespan [18,19]. These characteristics directly influence aquaculture productivity and reinforce the need to development sex-control technologies for this species. Although sex-specific molecular markers have been identified by our team (unpublished data) and the sex-determining gene (*amhY*) has been described by Pan et al. [20], important gaps remain regarding the genes, molecular pathways, and epigenetic mechanisms associated with sexual differentiation in *E. lucius*.

Given this, the present study performed whole-transcriptome sequencing of gonadal tissues from male and female *E. lucius* to identify sex-related candidate genes, characterize central regulatory pathways, and investigate transcriptional regulatory patterns associated with sexual differentiation. In addition to expanding knowledge about the molecular mechanisms involved in this process, these results provide theoretical support for future applications in sex-control technologies and in improving aquaculture production of this species.

## 2. Materials and Methods

### 2.1. Animals and Sample Collection

All experiments performed in this study were approved by the Animal Welfare and Ethics Committee of Xinjiang Agricultural University (Urumqi, China) under the approved protocol code 2022027. Three male and three female *E. lucius* (10 months old, sexually immature) with body length ranging from 37 to 43 cm and body weight from 478 to 503 g were selected as experimental animals in this study. Gonadal tissues were collected from each fish, immediately flash-frozen in liquid nitrogen, and subsequently transferred to an −80 °C ultra-low temperature freezer for storage.

### 2.2. Sex Identification of Experimental Fish

The *E. lucius* samples used in this study were the same individuals as in our previous work [21]. Briefly, six 10-month-old fish (three females and three males) were purchased from a local vendor. Genetic sex was determined using two primer sets: primer set 4 (amplifying a 606 bp band in females and both 606 bp and 320 bp bands in males) and *Amh* primers (amplifying a 500 bp band only in males). Phenotypic sex was confirmed by gonadal histology (paraffin sections). Only individuals with consistent genetic and phenotypic sex were included. For full details, see [21].

### 2.3. Construction and Sequencing of Whole Transcriptome Sequencing Libraries

#### 2.3.1. RNA Extraction and Quality Control

Total RNA was extracted from gonadal tissues using TRNzol Universal Reagent (TIANGEN, DP424, Beijing, China). RNA concentration was measured using a NanoDrop 2000 spectrophotometer (Thermo Scientific, Wilmington, DE, USA) and ranged from 242 to 1072 ng/μL. RNA integrity was assessed using an Agilent 2100 Bioanalyzer (Agilent Technologies, Santa Clara, CA, USA); all samples had RIN values ≥ 8.7, indicating high RNA quality.

#### 2.3.2. Library Preparation for mRNA, lncRNA, and circRNA

After passing quality inspection, RNA samples from gonads of 6 *E. lucius* individuals were subjected to library construction by Beijing Novogene Bioinformatics Technology Co., Ltd. (Beijing, China). For each individual, two types of libraries were constructed: (1) mRNA+lncRNA+circRNA combined library and (2) miRNA library, resulting in a total of 12 libraries (6 individuals × 2 library types). The library construction procedures for mRNA, lncRNA and circRNA were as follows. Qualified total RNA samples were subjected to differential pretreatment according to the molecular characteristics of the three RNA types. For circRNA enrichment, linear RNAs were digested using RNase R (Epicentre, RNR07250, Madison, WI, USA) to retain circular molecules. For lncRNA and mRNA enrichment, ribosomal RNA was removed using the TIANSeq rRNA Depletion Kit (TIANGEN, NR101-T1, Beijing, China) while maximally retaining poly(A)-tailed lncRNAs. Subsequently, fragmentation buffer was added to the two types of enriched products to fragment RNA into short fragments of 250–300 bp. Using the fragmented RNA as templates, first-strand cDNA was synthesized with reverse transcriptase from the Fast RNA-seq Lib Prep Kit V2 (RK20306, Wuhan, China) using random oligonucleotide primers (for circRNA) and random hexamer primers (for lncRNA+mRNA), respectively. Second-strand cDNA was then synthesized in a DNA polymerase I system using dNTPs containing dUTP as substrates. The synthesized double-stranded cDNA was purified, and then end repair, 3′-end A-tailing, and adapter ligation were performed using the same Fast RNA-seq Lib Prep Kit V2 (RK20306). Fragments of 370–420 bp were selected using AMPure XP beads, followed by PCR amplification to obtain circRNA libraries and lncRNA+mRNA strand-specific libraries. To minimize PCR bias, we used KAPA HiFi HotStart ReadyMix (Roche) with 12–15 cycles of amplification and employed dual-index primers. After passing quality inspection for integrity and insert size using a Fragment Analyzer 5300 (Agilent Technologies, Santa Clara, CA, USA) and effective concentration by qPCR using a QuantStudio 5 system (Applied Biosystems, Waltham, MA, USA), libraries were pooled according to their effective concentrations and the target sequencing data output, and high-throughput sequencing was performed using the Illumina NovaSeq 6000 platform (Illumina, San Diego, CA, USA) with paired-end sequencing strategy.

#### 2.3.3. Library Preparation for miRNA

miRNA library construction: 3′ and 5′ adapters (NEBNext^®^ Multiplex Small RNA Library Prep Set, New England Biolabs, Ipswich, MA, USA) were ligated to the 3′ and 5′ ends of small RNAs respectively for specific labeling. Subsequently, reverse transcription primers (adapter-specific primers complementary to the 3′ adapters; synthesized by Invitrogen, Waltham, MA, USA) were added to hybridize with the adapter-ligated small RNAs and first-strand cDNA was synthesized using the Fast RNA-seq Lib Prep Kit V2 (RK20306), followed by PCR amplification using KAPA HiFi HotStart ReadyMix (Roche, Basel, Switzerland) to generate double-stranded cDNA libraries. After purification, libraries with insert sizes between 18–40 bp were selected.

#### 2.3.4. Quality Control and Sequencing

Following quality inspection using the same Fragment Analyzer 5300 (Agilent Technologies) and qPCR (QuantStudio 5, Applied Biosystems), qualified libraries were pooled according to their effective concentrations and the target sequencing data output, and high-throughput sequencing was performed using the Illumina NovaSeq 6000 platform (Illumina, San Diego, CA, USA) with a paired-end 150 bp (PE150) strategy. The sequencing data output was >12 Gb per sample for all libraries.

### 2.4. Quality Control and Differentially Expressed Gene Analysis

Raw sequencing data were subjected to quality control (QC) prior to subsequent bioinformatics analysis. Specifically, low-quality bases were defined as those with a Phred quality score ≤ 5 and low-quality reads (with ≥50% low-quality bases), reads containing adapter contamination, and reads with more than 10% N bases were filtered out to obtain clean reads. For mRNA and lncRNA analyses, clean reads were aligned to the published *E. lucius* reference genome (GCF_011004845.1) using Hisat2 software (version 2.0.5; https://daehwankimlab.github.io/hisat2/, accessed on 4 June 2025). The corresponding gene annotation file (GTF format, NCBI RefSeq release 104) was used for read quantification. Gene-level read counts were quantified using featureCounts (version 2.0.1; https://subread.sourceforge.net/, accessed on 4 June 2025). For circRNA identification, unmapped reads from the Hisat2 alignment were subjected to CIRI2 (version 2.0.6; https://ciri-cookbook.readthedocs.io/en/latest/CIRI2.html, accessed on 4 June 2025) to detect back-spliced junction reads. Only circRNAs supported by at least two unique back-spliced reads were retained for further analysis. This reference genome was assembled by the Vertebrate Genomes Project (G10K-VGP) using a combination of PacBio long reads, Bionano optical maps, 10X Genomics linked reads, Arima Genomics Hi-C, and Illumina short reads. The assembly is at the chromosome level (25 chromosomes, scaffold N50 = 37.47 Mb, total length ~918 Mb), and BUSCO completeness against the eukaryota_odb10 dataset reached 96.20%, indicating high assembly integrity and gene space coverage. Therefore, this high-quality reference genome is well-suited for identifying lncRNAs and circRNAs, which rely on accurate detection of spliced junctions. Subsequently, expression quantification was performed on the aligned, assembled and filtered transcripts as well as the predicted transcripts: For visualization purposes, the expression levels of lncRNAs and mRNAs were normalized to FPKM (Fragments Per Kilobase of transcript per Million mapped reads), and differential expression significance analysis was performed using the edgeR package (version 3.32.1; https://bioconductor.org/packages/release/bioc/html/edgeR.html, accessed on 4 June 2025). For known and novel microRNAs (miRNAs) and circular RNAs (circRNAs) in each sample, raw read counts were obtained. For visualization purposes only, expression levels were normalized to TPM (Transcripts Per Million). Differential expression analysis between female and male groups was performed on raw read counts using the DESeq2 package (version 1.34.0; https://bioconductor.org/packages/release/bioc/html/DESeq2.html, accessed on 4 June 2025) with its default median-of-ratios normalization. Differentially expressed genes were screened with the criteria of |log_2_ fold change| > 1 and FDR (Benjamini–Hochberg adjusted *p*-value) < 0.05. Genes with average counts per million (CPM) < 1 in all samples were filtered out prior to analysis as lowly expressed transcripts. Multiple testing correction was performed using the Benjamini–Hochberg procedure to control the false discovery rate (FDR).

### 2.5. Functional Enrichment Analysis of DEGs

To gain insights into the biological functions of differentially expressed genes (DEGs), we performed GO (Gene Ontology) and KEGG (Kyoto Encyclopedia of Genes and Genomes) enrichment analyses. GO analysis aims to identify enriched biological processes, cellular components, and molecular functions among the DEGs, thereby revealing their potential roles in physiological processes. KEGG pathway analysis was used to identify the major metabolic and signaling pathways involving the DEGs. These analyses help link DEGs to specific biological functions and pathways, providing a functional annotation basis for subsequent discussions on the molecular mechanisms of sexual differentiation. Gene Ontology (GO) term enrichment analysis was performed using the R package GOseq (version 1.44.0; https://bioconductor.org/packages/release/bioc/html/goseq.html, accessed on 4 June 2025), with a significance threshold of adjusted *p*-value < 0.05. Kyoto Encyclopedia of Genes and Genomes (KEGG) pathway enrichment analysis was performed using KOBAS software (version 3.0; http://kobas.cbi.pku.edu.cn/kobas3/, accessed on 4 June 2025).

### 2.6. Construction of ceRNA Regulatory Networks

The interactions between lncRNAs and miRNAs were predicted using miRanda (version 3.3a; https://cbio.mskcc.org/microrna_data/manual.html, accessed on 4 June 2025), and the miRNA-mRNA target relationships were predicted in combination with miRDB (https://mirdb.org/, accessed on 4 June 2025) and TargetScan (version 8.0; https://www.targetscan.org/vert_80/, accessed on 4 June 2025) Core interacting pairs from the two modules were integrated to construct the ceRNA network, and visualization and topological analysis were performed using Cytoscape (version 3.6.0; https://cytoscape.org/, accessed on 4 June 2025) to illustrate the roles of the regulatory axes.

## 3. Results

### 3.1. Sex Identification Results of Experimental Fish

As previously reported [21], all three genetically female individuals showed the expected single 606 bp band with primer set 4 and no Amh product, while males showed both bands (606 bp and 320 bp) and a 500 bp Amh band (see [21] for original gel images). Gonadal histology confirmed the presence of ovaries in females and testes in males.

### 3.2. Summary of Transcriptome Sequencing Data

High-throughput sequencing was performed on whole-transcriptome libraries from female and male gonads, resulting in the construction of 6 sequencing libraries. A total of 572 million raw reads were generated from ovarian and testicular tissues (Table 1). After removing low-quality reads and reads containing adapter sequences, 562 million high-quality clean reads were obtained. These clean reads were successfully aligned to the high-quality reference genome of *E. lucius*, with alignment rates ranging from 87.82% to 96.42% for individual samples. The Q20 base percentages were all above 99.23%, the Q30 base percentages were all above 96.69%, and the GC contents ranged from 44.35% to 48.69%. These results indicated that the sequencing data were of high quality and suitable for subsequent analyses.

### 3.3. Differential Gene Expression Analysis of Gonadal Tissues

**Figure 1:**
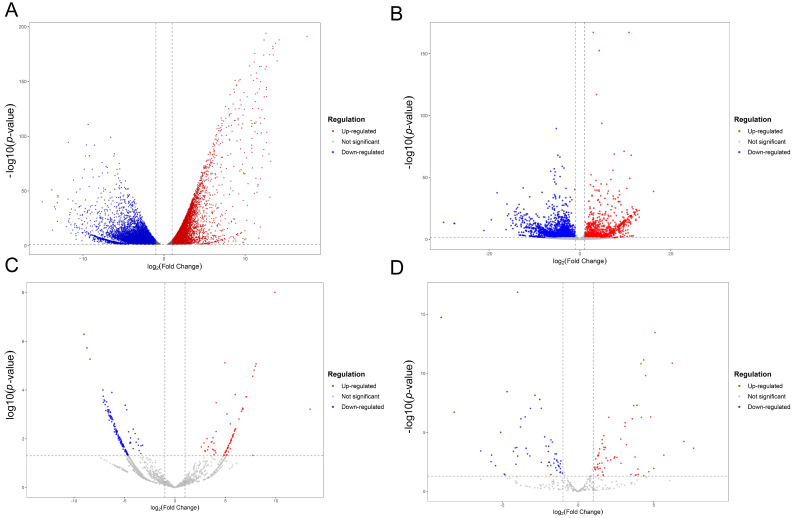
Volcano plots of differentially expressed RNAs. (**A**) Volcano plot of DEmRNAs; (**B**) Volcano plot of DELncRNAs; (**C**) Volcano plot of DEcircRNAs; (**D**) Volcano plot of DEmiRNAs. Note: Gray dots represent non-significant genes, red dots represent up-regulated DEGs, and bule dots represent down-regulated DEGs.

Differential expression (DE) analysis of genes between female and male gonads was performed. The results showed that 14,941 DE mRNAs (6808 upregulated and 8133 downregulated) were identified (Figure 1A); 2055 DE lncRNAs (663 upregulated and 1392 downregulated) were identified (Figure 1B); 229 DE circRNAs (84 upregulated and 145 downregulated) were identified (Figure 1C); 119 DE miRNAs (62 upregulated and 57 downregulated) were identified (Figure 1D).

### 3.4. GO Functional Annotation and KEGG Enrichment Analysis of Differentially Expressed Transcripts

#### 3.4.1. GO Functional Annotation and KEGG Enrichment Analysis of DEmRNAs

**Figure 2:**
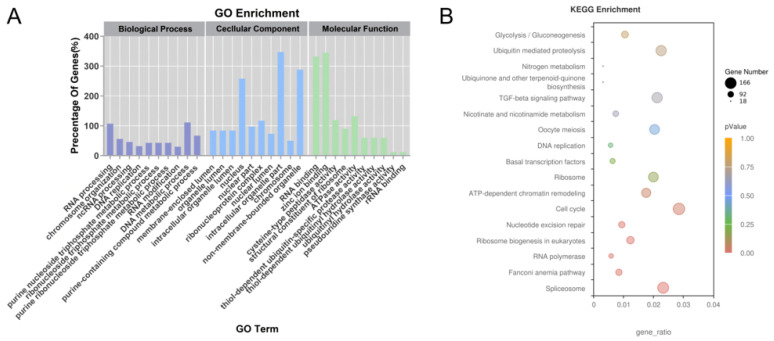
GO Functional Annotation and KEGG Enrichment Analysis of Target Genes of DEmRNAs. (**A**) GO Functional Annotation Analysis of Target Genes of DEmRNAs; (**B**) KEGG Enrichment Analysis of Target Genes of DEmRNAs.

GO enrichment analysis of differentially expressed mRNAs (Figure 2) indicated greater representation in biological processes related to RNA processing, gene expression regulation, and nucleic acid metabolism. In cellular components, genes were mainly associated with ribonucleoprotein complexes, intracellular organelles, and membrane-bound organelles. In molecular functions, binding to RNA, nucleic acids, zinc ions, and proteins stood out. KEGG analysis revealed enrichment in pathways (Figure 2) such as alternative mRNA splicing (spliceosome), ubiquitin-mediated proteolysis, TGF-β signaling, oocyte meiosis, and cell cycle. The integration of these results allowed the identification of candidate genes associated with development and sexual differentiation, including *FANCL*, *DDX5*, and *SRSF5B*, which are upregulated in females, and *STAR*, *FDX1B*, and *ITGA2B*, which are upregulated in males (Table 2).

#### 3.4.2. GO Functional Annotation and KEGG Enrichment Analysis of Target Genes of DElncRNAs

**Figure 3:**
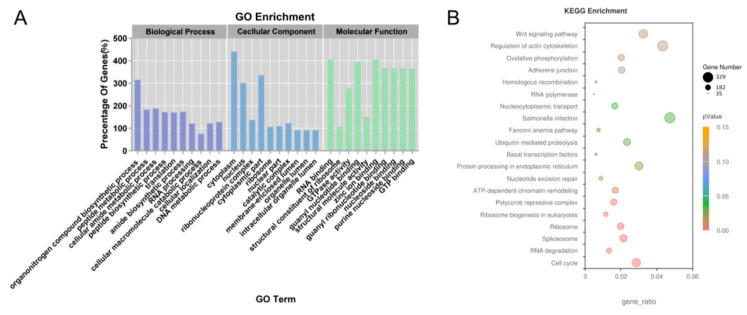
GO Functional Annotation and KEGG Enrichment Analysis of Target Genes of DElncRNAs. (**A**) GO Functional Annotation Analysis of Target Genes of DElncRNAs; (**B**) KEGG Enrichment Analysis of Target Genes of DElncRNAs.

GO functional enrichment analysis (Figure 3) of target genes of DElncRNAs revealed a predominance in biosynthetic processes of nitrogenous organic compounds and translation. In cellular components, the genes were mainly associated with the cytoplasm and ribonucleoprotein complexes. In molecular functions, RNA binding, a structural constituent of the ribosome, GTPase activity, and zinc ion binding stood out. KEGG analysis indicated enrichment in pathways (Figure 3) such as Wnt signaling, cell cycle, ubiquitin-mediated proteolysis, regulation of the actin cytoskeleton, and spliceosome. Among the differentially expressed lncRNAs, TCONS_00090565, TCONS_00071180, and TCONS_00069127 showed overexpression in females, while TCONS_00049539, TCONS_00043965, and TCONS_00050262 were overexpressed in males. Their target genes were associated with pathways related to development, sex determination, and sexual differentiation.

#### 3.4.3. GO Functional Annotation and KEGG Enrichment Analysis of Host Genes of DEcircRNAs

**Figure 4:**
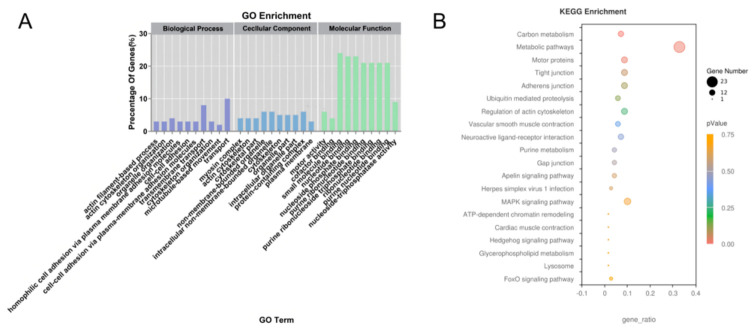
GO Functional Annotation and KEGG Enrichment Analysis of Target Genes of DEcircRNAs. (**A**) GO Functional Annotation Analysis of Target Genes of DEcircRNAs; (**B**) KEGG Enrichment Analysis of Target Genes of DEcircRNAs.

GO functional enrichment analysis (Figure 4) of host genes of DEcircRNAs indicated a predominance of activities related to cell motility, including actin binding, motor activity, and actin filament-based processes. Enrichments were also observed in binding to small molecules, nucleotides, and purine ribonucleoside triphosphates, as well as ATPase activity. In cellular components and biological processes, the cytoskeleton, actin complex, homophilic cell adhesion, and microtubule-based movement stood out. In the KEGG analysis (Figure 4), the host genes were enriched in metabolic pathways, motor proteins, regulation of the actin cytoskeleton, purine metabolism, ubiquitin-mediated proteolysis, and MAPK, Hedgehog, FoxO, and Apelin signaling pathways. Among the differentially expressed circRNAs, circ_03442 and circ_05641 showed overexpression in females, while circ_02857 and circ_04300 were overexpressed in males. The host genes associated with these circRNAs were enriched in pathways related to development, sex determination, and sexual differentiation.

#### 3.4.4. GO Functional Annotation and KEGG Enrichment Analysis of Target Genes of DEmiRNAs

**Figure 5:**
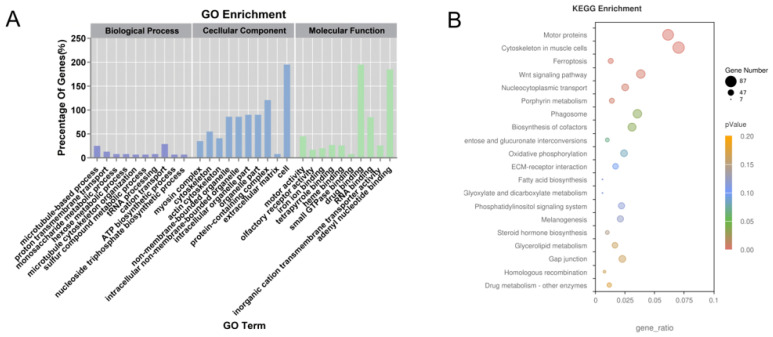
GO Functional Annotation and KEGG Enrichment Analysis of Target Genes of DEmiRNAs. (**A**) GO Functional Annotation Analysis of Target Genes of DEmiRNAs; (**B**) KEGG Enrichment Analysis of Target Genes of DEmiRNAs.

GO functional enrichment analysis (Figure 5) of target genes of DEmiRNAs revealed a predominance of activities related to cell motility and interaction with the cytoskeleton, including actin binding, motor activity, and ATPase activity. Enrichments were also observed in binding to nucleotides, purine ribonucleoside triphosphates, small molecules, and nucleic acids. In cellular components and biological processes, the actin complex, cytoskeleton, intracellular and membrane-bound organelles, cytoplasm, plasma membrane, actin filament-based processes, cell–cell adhesion, and substance transport stood out. In the KEGG analysis (Figure 5), the target genes were enriched in pathways associated with motor proteins, regulation of the actin cytoskeleton, Wnt signaling, steroid hormone biosynthesis, porphyrin and purine metabolism, glycolysis/gluconeogenesis, pentose phosphate pathway, glycerophospholipid metabolism, fatty acid biosynthesis, receptor–extracellular matrix interaction, and nucleocytoplasmic transport. Among the differentially expressed miRNAs, dre-miR-204-5p, dre-miR-192, and dre-miR-194a showed overexpression in females, while dre-miR-23b-5p, dre-miR-125b-2-3p, and dre-miR-727-5p were overexpressed in males. The target genes of these miRNAs were enriched in the aforementioned pathways, suggesting that these six miRNAs may act in the regulation of gonadal development in *E. lucius* through these signaling networks.

#### 3.4.5. Construction of Sex-Related ceRNA Regulatory Networks

**Figure 6:**
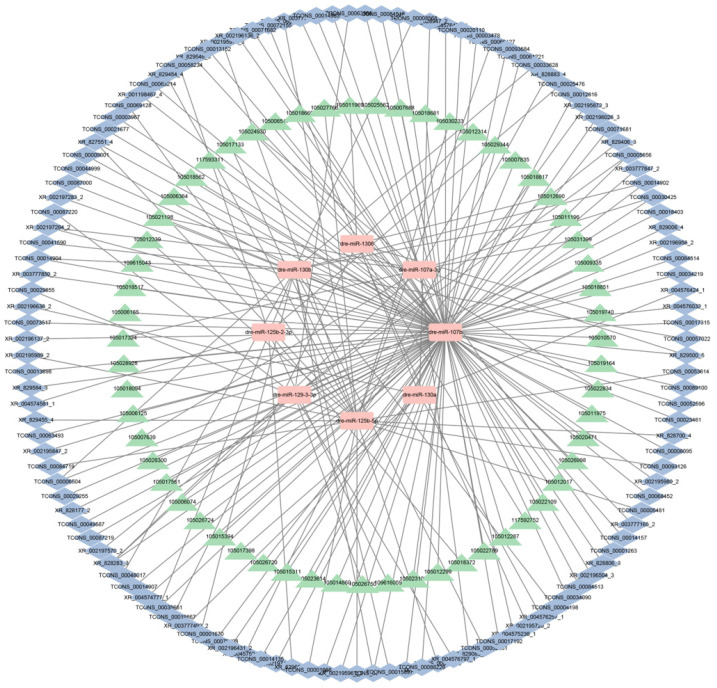
lncRNA–miRNA–mRNA network. Note: Different color hues represent lncRNAs (blue), mRNAs (green) and miRNAs (pink).

By integrating the analysis results of DE miRNA-DE mRNA and DE lncRNA-DE mRNA interaction networks (Figure 6), this study successfully identified a set of DEmRNAs co-regulated by multiple miRNAs and lncRNAs.

## 4. Discussion

Molecular and histological validation consistently confirmed sexual dimorphism in *E. lucius*, with females exhibiting ovaries and absence of the *Amh* gene, while males presented testes and presence of *Amh*, reinforcing the role of this gene as a central marker and regulator of male gonadal differentiation in teleosts. Sequencing data showed high quality, with high alignment rates to the reference genome and Q20/Q30 values greater than 99% and 96%, respectively, demonstrating the reliability of subsequent analyses. Differential analysis identified a broad set of differentially expressed transcripts between the sexes, including 14,941 mRNAs, 2055 lncRNAs, 229 circRNAs, and 119 miRNAs, evidencing that gonadal differentiation in *E. lucius* involves a complex regulatory network composed of coding and non-coding RNAs.

Among the differentially expressed mRNAs, several genes previously associated with sex determination and differentiation in teleosts showed sexually dimorphic expression in *E. lucius*. In females, the *FANCL*, *DDX5*, and *SRSF5B* genes exhibited significantly higher expression levels. *FANCL*, a central component of the DNA repair pathway associated with Fanconi anemia, plays an essential role in female sexual differentiation. In zebrafish, mutations in this gene can induce sex reversal from females to males [22], reinforcing its importance for female gonadal development. The ATP-dependent RNA helicase *DDX5* also plays a fundamental role in female sexual differentiation and oocyte maturation. In zebrafish, most individuals with a deletion of the *ddx5* gene develop as fertile males [23], indicating that this gene is crucial for the establishment and maintenance of the female gonadal pathway. In turn, *SRSF5B*, a member of the serine/arginine-rich splicing factor family, can modulate estrogen signaling and gonadal development through the regulation of RNA splicing [24]. Furthermore, homologs of the SRSF family have been described as regulators of splicing of genes associated with reproduction in the semi-smooth-tongued flounder [25], suggesting a possible similar conserved function in *E. lucius*.

In males, the genes *STAR*, *FDX1B*, and *ITGA2B* showed significantly elevated expression. *STAR* acts as the main rate-limiting factor for steroid hormone synthesis. High testicular expression of *StAR* is closely associated with testicular development and spermatogenesis in the semi-smooth tongue flounder [26] and *Nile tilapia* [27]. The *FDX1B* gene, an important electron-donating cofactor for mitochondrial steroidogenic enzymes, participates in the synthesis of androgens and glucocorticoids, in addition to acting in the regulation of male gonadal differentiation and spermatogenesis [28]. In zebrafish, knockout of *fdx1b* results in reduced androgen secretion, compromised testicular structure, and even the emergence of feminized phenotypes [29].

The *ITGA2B* integrin gene is involved in cell adhesion, proliferation, and differentiation processes, and may contribute to testicular development and the maintenance of the sexual phenotype [30]. This gene has been identified as a transcript preferentially expressed in males in the gonadal transcriptomes of rainbow trout (*Oncorhynchus mykiss*) and Atlantic salmon (*Salmo salar*) [31,32]. Furthermore, studies with other members of the integrin family reinforce its possible role in fish reproduction. In gilthead seabream (*Sparus aurata*), for example, the β1 isoform of the integrin, *ITGB1b*, showed specific expression in testes and brain, with a peak during spermatogenesis and a positive correlation with testosterone levels, indicating its involvement in testicular development [33]. Taken together, these results suggest that members of the integrin family play conserved roles in gonadal differentiation and spermatogenesis in teleosts, strengthening the hypothesis that *ITGA2B* also participates in these processes in *E. lucius*.

Functional enrichment analyses by GO and KEGG of DEGs highlighted processes related to RNA binding and processing, spliceosome, cell cycle, ubiquitin-mediated proteolysis, TGF-β signaling, and oocyte meiosis as central pathways associated with sexual differentiation in *E. lucius*. These findings are in agreement with previous transcriptomic studies in teleosts, which demonstrate that post-transcriptional regulatory mechanisms, especially alternative splicing, as well as post-translational regulatory processes, such as protein ubiquitination, play essential roles in sexual determination and differentiation [14,25,34,35].

Differentially expressed lncRNAs, circRNAs and miRNAs also showed enrichment in RNA binding, nucleic acid binding, cytoskeleton remodelling, cell adhesion, and metabolic pathways. Previous studies demonstrate that lncRNAs can regulate RNA processing and translation through interactions mediated by RNA-binding proteins [36,37]. Similarly, circRNAs have been associated with the regulation of metabolic pathways, MAPK signalling, and actin cytoskeleton dynamics [38,39]. miRNAs modulate the Wnt signalling pathway (medaka), steroid hormone biosynthesis (*Nile tilapia*), and germ cell migration (zebrafish) [40,41,42]. Taken together, these results reinforce the idea that non-coding RNAs exert multilevel regulatory control over gonadal differentiation in *E. lucius*, acting in an integrated manner on transcriptional, post-transcriptional, and cellular processes essential for sexual development.

Finally, we constructed a ceRNA regulatory network involving lncRNA–miRNA–mRNA interactions, in which dre-miR-107b showed the highest node connectivity, suggesting a possible central role in the regulation of gonadal differentiation. Members of the miR-107 family have already been described with sexually dimorphic expression in different vertebrates. In chickens, miR-107 acts directly on *NR5A1* and *CYP19A1*, participating in the regulation of ovarian development [43]. In zebrafish, dre-miR-107b has been identified in gonadal tissues and associated with gonadal development through the regulation of downstream target genes [44]. Furthermore, in mammals, miR-107 homologs have also been linked to testicular development and spermatogenesis [45,46]. This evidence of conservation across species further reinforces the hypothesis that dre-miR-107b may have important functions in the sexual differentiation of fish.

However, a limitation of this study is the lack of cross-species conservation analysis specifically for dre-miR-107b in *E. lucius*. Due to the incomplete annotation of this species’ genome, the prediction of target genes across species based on sequence alignment has low reliability. Thus, although dre-miR-107b exhibited sexually dimorphic expression in our data, this pattern cannot be directly interpreted as evidence of functional conservation.

Future studies should prioritize improving the genomic annotation of *E. lucius*, as well as conducting comparative genomic analyses with phylogenetically related species, such as salmonids and other esocids. Furthermore, experimental approaches, including dual luciferase reporter assays, will be fundamental to validating the predicted regulatory interactions. Even so, the other non-coding RNAs identified in this study as potential regulators of genes associated with sexual differentiation represent promising candidates for future investigations, and may play important roles in the regulation of gonadal development and sexual differentiation in *E. lucius*.

## 5. Conclusions

This study presents the first complete transcriptome data of the male and female gonads of *E. lucius*, identifies key genes and pathways with differential expression between the sexes, and constructs lncRNA–miRNA–mRNA networks centered on dre-miR-107b, establishing a theoretical basis for elucidating the mechanism of sexual differentiation in this species. Taken together, these results broaden the knowledge about the molecular mechanisms of sexual differentiation in *E. lucius* and provide an important basis for future functional studies in the species.

## Figures and Tables

**Table 1:** Whole-transcriptome raw data. Note: F1–3 represents females, M1–3 represents males.

Sample	Raw Reads	Clean Reads	Mapped Reads	Error_Rate	GC Content	% ≥ Q20	% ≥ Q30
F1	96,841,952	95,221,924	91,596,484 (96.19%)	0.01	48.67	99.35	96.95
F2	93,521,852	92,155,308	88,858,855 (96.42%)	0.01	48.69	99.38	96.95
F3	92,838,244	90,798,446	87,220,368 (96.06%)	0.01	48.69	99.23	96.69
M1	99,367,692	97,498,310	85,624,537 (87.82%)	0.01	44.35	99.3	96.93
M2	96,473,368	94,810,330	88,023,892 (92.84%)	0.01	45.68	99.25	96.8
M3	93,226,608	91,614,674	81,826,076 (89.32%)	0.01	44.86	99.3	96.97

**Table 2:** DEmRNAs associated with sex differentiation in *E. lucius* gonads. Note: F: female; M: male; FPKM values are means of three biological replicates per group.

Gene Name	log_2_FC	FDR	Expression Level (FPKM)	Functional Annotation
*FANCL*	2.128	3.026 × 10^−8^	15.45 (F)/3.14 (M)	E3 ubiquitin–protein ligase
*DDX5*	2.187	1.109 × 10^−11^	422.2 (F)/82.2 (M)	ATP-dependent RNA helicase (DEAD-box)
*SRSF5B*	1.196	3.625 × 10^−5^	22.62 (F)/6.53 (M)	Serine/arginine-rich splicing factor
*STAR*	−8.855	2.204× 10^−68^	0.12 (F)/51.96 (M)	Steroidogenic acute regulatory protein
*FDX1B*	−6.007	7.316 × 10^−9^	0.11 (F)/6.70 (M)	Ferredoxin (electron transfer for steroid synthesis)
*ITGA2B*	−3.773	6.824 × 10^−11^	0.30 (F)/3.70 (M)	Integrin subunit alpha 2b (cell adhesion)

## Data Availability

The results of whole-genome methylation sequencing of *Esox lucius.* have been deposited in the BioProject database of the National Center for Biotechnology Information (NCBI) with the accession number PRJNA1455254, and the public access URL is: https://www.ncbi.nlm.nih.gov/bioproject/PRJNA1455254 (accessed on 19 April 2026).
